# Steap4 attenuates high glucose and S100B-induced effects in mesangial cells

**DOI:** 10.1111/jcmm.12472

**Published:** 2015-03-27

**Authors:** Chao-Tang Chuang, Jinn-Yuh Guh, Chi-Yu Lu, Yeng-Tseng Wang, Hung-Chun Chen, Lea-Yea Chuang

**Affiliations:** a Graduate Institute of Medicine, College of Medicine, Kaohsiung Medical UniversityKaohsiung, Taiwan; b Department of Internal Medicine, College of Medicine, Kaohsiung Medical UniversityKaohsiung, Taiwan; c Department of Internal Medicine, Kaohsiung Medical University Hospital, Kaohsiung Medical UniversityKaohsiung, Taiwan; d Department of Biochemistry, College of Medicine, Kaohsiung Medical UniversityKaohsiung, Taiwan; e Center for Lipid and Glycomedicine Research, Kaohsiung Medical UniversityKaohsiung, Taiwan

**Keywords:** Steap4, S100B, high glucose, protein interaction

## Abstract

Six-transmembrane epithelial antigen of prostate 4 (Steap4)-knockout mice develop hyperglycaemia and inflammation whereas Steap4 overexpression attenuates atherosclerosis in diabetic mice. Thus, we studied the roles of Steap4 in high glucose (HG, 27.5 mM) or S100B (1 μM, a ligand for the receptor for advanced glycation end-product or RAGE)-induced effects in mouse mesangial (MES13) cells. We found that HG-induced Steap4 protein expression was dependent on S100B. HG increased cell membrane, but not cytosolic, Steap4 protein expression. HG increased protein-protein interaction between Steap4 and S100B, which was confirmed by mass spectrometry of immunoprecipitated S100B. SP600125, LY294002 and AG490 attenuated S100B-induced Steap4 protein expression or gene transcriptional activity. A mutation in signal transducer and activator of transcription 3 (Stat3) site 2 of the Steap4 promoter constructs resulted in a marked decrease in HG or S100B-induced activation of Steap4 gene transcription. Overexpression of Steap4 attenuates HG or S100B-induced collagen IV, fibronectin and cyclooxygenase 2 protein expression. Overexpression of Steap4 attenuates HG or S100B-induced transforming growth factor-β (TGF-β). Moreover, overexpression of Steap4 attenuates S100B-induced signalling. Finally, overexpressing Steap4 attenuated renal expression of fibronectin, S100B, TGF-β, type IV collagen, p-Akt, p-extracellular signal regulated kinase 1/2 and p-Stat3 in streptozotocin-diabetic mice. Thus, overexpression of Steap4 attenuated HG or S100B-induced effects in MES13 cells and attenuated some of S100B-induced effects in diabetic mouse kidneys.

## Introduction

Diabetic nephropathy (DN) is associated with many factors including angiotensin II (Ang II), receptor for advanced glycation end-products (RAGE), transforming growth factor-β (TGF-β) and inflammation 1,2. Among them, inhibitors of Ang II signalling are current standards of therapy for DN, albeit with significant residual risk of progression 3.

Among the many RAGE ligands 4, S100B is increased in diabetic glomeruli 5 and binds to RAGE with a high affinity similar to that of carboxy-methyl-lysine-AGE 6. S100B induces TGF-β and fibronectin while activating extracellular signal-regulated kinase (ERK1/2) and p38 kinase in mesangial cells 7. Moreover, S100B is the only RAGE ligand which universally induces inflammatory cytokines 8. Interestingly, S100B can also be a RAGE-independent intracellular regulator 9.

Six-transmembrane epithelial antigen of prostate 4 [Steap4, also known as six transmembrane protein of prostate 2 (STAMP2) or tumour necrosis-α (TNFα)-induced adipose-related protein (TIARP)] is an anti-inflammatory protein 10,11 induced by inflammatory signals such as TNFα and interleukin-6 (IL6) 12,13. Interestingly, Steap4-knockout mice develop insulin resistance, hyperglycaemia and inflammation 14.

Steap4 colocalizes with caveolin-1 in cell membrane caveolae 15, a place where RAGE resides 16,17. Thus, it is interesting to study the interactions between Steap4 and these cell surface receptors. Additionally, because high glucose (HG) increases IL6 only in Steap4-deficient [but not wild-type (WT)] adipocytes 14 while overexpressing Steap4 suppresses atherosclerosis in diabetic mice 18, we hypothesize that Steap4 overexpression may attenuate HG or S100B-induced effects in mesangial cells.

Thus, our purpose is to study the roles of Steap4 in HG or S100B-induced effects in mesangial cells. Moreover, we also study the interaction between Steap4 and S100B and the effects of overexpressing Steap4 on the streptozotin-diabetic mice.

## Materials and methods

### Materials

Cell culture reagents were purchased from the Gibco Co. (Grand Island, NY, USA). Dimethyl sulphoxide, PD98059 [an extracellular regulated kinase (ERK1/2) inhibitor], SB203580 (a p38 kinase inhibitor), SP600125 [a Jun kinase (JNK) inhibitor], SB431542 (a type I TGF-β receptor inhibitor), LY294002 [a phosphoinositide-3 kinase (PI3K) inhibitor] and AG490 [a Janus kinase (JAK2) inhibitor] were purchased from the Sigma-Aldrich Co. (St. Louis, MO, USA). S100B was purchased from the Abcam Co. (Cambridge, UK).

The antibodies used were: S100B, collagen IV (col4α1), cyclooxygenase 2 (COX2), pan-cadherin (Abcam); Steap4 (Novus Biologicals, Littleton, CO, USA), fibronectin and RAGE (Chemicon, Temecula, CA, USA), ERK1/2, p-ERK1/2 (Thr202/Tyr204), p38 kinase, p-p38 kinase (Thr180/Try182), Akt, p-Akt (Thr308), Signal transducer and activator of transcription 3 (Stat3), p-Stat3 (Tyr705), Smad2/3, p-Smad2/3 (Ser433/435) (Cell Signaling Technology Inc., Danvers, MA, USA), GAPDH (Santa Cruz Biotechnology, Santa Cruz, CA, USA) and α-tubulin (Thermo Scientific, Fremont, CA, USA).

### Cells

Mouse mesangial cell line (MES13 cells, CRL-1927) was purchased from the Bioresource Collection and Research Center (Hsinchu, Taiwan). Cells were cultured in 3:1 mixture of DMEM and Ham's F12 medium (glucose 6.67 mM), 14 mM HEPES, 1% penicillin/streptomycin and 5% fetal bovine serum (FBS) in 5% CO_2_ at 37°C. MES13 cells were serum-starved for 16 hrs before being treated with 5% FBS-containing HG, S100B (1 μM) or the signalling pathway inhibitors.

### Extraction of membrane protein

Briefly, cells were pelleted and the cytosolic and membrane protein were fractionated by the CNMCS compartmental protein extraction kit (BioChain Institute, Newark, CA, USA) according to the manufacturer's instructions and then lysates in cell membrane and cytosolic fractions are measured by immunoblotting. Pan-cadherin was used as a cell membrane marker.

### Immunoprecipitation

Briefly, proteins from MES13 cells lysed in the lysis buffer were incubated with antibodies (S100B, Steap4, RAGE) and protein A/protein G magnetic beads (Millipore Corp., Billerica, MA, USA). Binding reactions were for 5 hrs at 4°C with continual rotation. The beads were collected and washed three times (3 min./wash) with the lysis buffer. Bound proteins were eluted by boiling in laemmli sample buffer. Immunoprecipitated proteins were separated by SDS-PAGE and immunoblotted with antibodies to their interaction partners.

### Immunoblotting

Proteins extracted from cells were lysed and separated by SDS-PAGE and transfer to polyvinylidene difluoride membranes. After 5% non-fat milk blocking, blots were incubated with the primary antibodies in blocking solution for 1 hr followed by two 5-min. washes in PBS containing 0.1% Tween. The membranes were then incubated with horseradish peroxidase-conjugated secondary antibodies for 30 min. Enhanced chemiluminescence reagents were employed to depict the protein bands on membranes. Results were expressed as the ratio of intensity of the protein of interest to that of α-tubulin or the indicated protein from the same sample.

### Plasmids

The pCMV-SPORT6-Steap4 expression plasmid and the pCMV-SPORT6 vector were purchased from Life Technologies Corp. (Carlsbad, CA, USA). The p-1402 porcine Steap4 promoter plasmid, in the pGL3-Basic vector (Promega Corp., Madison, WI, USA), was a gift of Dr. Chen 19. The WT (−757 to +26 bp) pGL3-mouse Steap4 promoter and the mutant [Mut S1 (−230 bp, STAT3 site 1), Mut S2 (−116 bp, STAT3 site 2), Mut S3 (−52 bp, STAT3 site 3), Mut S1.S2, Mut S1.S3, Mut S2.S3 and Mut S1.S2.S3] plasmids were gifts of Dr. Hollenberg 20. The TGF-β bioactivity reporter p3TP-lux was a gift of Dr. Massagué 21 and the TGF-β promoter plasmid phTG5-luc was a gift of Dr. Virelizier 22.

### Transient transfection

Plasmids were transiently transfected into MES13 cells in 6-well plates (1 × 10^4^ cells/well) by using the TurboFect reagent (Thermo Scientific). Medium containing 5% FBS was added 24 hrs later and cells were treated with HG or 1 μM S100B for the indicated times. Cells were lysed and luciferase activity was measured by the Dynatech ML1000 luminometer (Dynatech Laboratories, Inc., Chantilly, VA, USA).

### Liquid chromatography-mass spectrometry

Briefly, cellular protein (1 μg/μl) was mixed with 100 μl acetone and centrifuged at 15,700 g for 10 min. After centrifugation, the supernatant was discarded and the protein residues were kept and evaporated to dry. Protein residues were re-dissolved with 18 μl 25 mM ammonium bicarbonate aqueous solution, reduced and alkylated, and then sequence-grade trypsin (Promega) 2 μl was added and digested at 37°C for 16 hrs. After digestion, tryptic peptide solution was injected into the nano LC system, precursor ions of peptides were fragmented by collision gas to obtain tandem MS (MS/MS) spectra and detected by the high resolution linear ion-trap (LTQ) Orbitrap mass spectrometer (Thermo Fisher Scientific Inc., Waltham, MA, USA). Raw data files (which contained precursor and fragment ions) were processed with Mascot Distiller software (Matrix Science Inc., Boston, MA, USA) to create the peak lists which were uploaded to the Mascot server (Matrix Science Inc.) for protein identification.

### Streptozotocin-diabetic mice

Male ICR mice (6 weeks of age) were purchased from BioLASCO Taiwan Co. (Taipei, Taiwan) and fed *ad libitum*. Four male ICR mice were given intraperitoneal injection of sodium citrate buffer (control, *N* = 4). After fasting for 6 hrs, diabetes was induced by an intraperitoneal injection of 55 mg/kg STZ (Sigma-Aldrich Co., St Louis, MO) in sodium citrate buffer (10 mM, pH 4.5) for five consecutive days (*N* = 24) 23. These mice had blood glucose levels of more than 22.2 mmol/l 1 week after the completion of streptozotocin injection. Lantus insulin (Sanofi Aventis, Paris, France) was given subcutaneously to keep blood glucose levels to be less than 27.8 mmol/l.

Gene therapy was achieved by weekly intravenous injection of 50 μg plasmids (with Turbofect *in vivo* Transfection Reagent, Thermo Scientific) *via* the tail vein 24. Eight diabetic mice were given weekly intravenous injection of the pCMV-SPORT6 empty plasmid once they had blood glucose levels of more than 22.2 mmol/l. Eight diabetic mice were given weekly intravenous injection of the pCMV-SPORT6-Steap4 expression plasmid at the same time. Mice were anesthetized with Zoletil (Virbac Taiwan Co., Ltd., Taipei, Taiwan) on week 8, perfused and kidneys were removed, immersed in 4% paraformaldehyde and kidney slices were embedded in the paraffin block and cut into 3-μm sections for immunohistochemical study after microwave treatment and blockade of non-specific responses 25. All animal procedures were approved and done in accordance with the national guidelines and the guidelines by the Kaohsiung Medical University Animal Experiment Committee which were equivalent to the NIH Guide for the Care and Use of Laboratory Animals.

### Immunohistochemistry

Briefly 25, paraffin sections were de-paraffinized and rehydrated. The sections were incubated with primary antibodies: Steap4 (Novus Biologicals), S100B, TGF-β, col4α1, COX2 (Abcam), p-ERK1/2, p-Akt, p-Stat3 (Cell Signaling Technology Inc.) and secondary antibodies, stained by Dako REAL™ EnVision™ (Detection System Peroxidase/DAB+, Dako Corp., Carpinteria, CA, USA), counterstained with haematoxylin. The extent of immunostaining was determined in each mouse in 20 glomeruli at 400× magnification.

### Statistical analysis

The values were expressed as the mean ± SEM. *In vitro* experimental data were collected from at least three repeated experiments. Unpaired Student's *t*-tests were used for the comparison between two groups. *P* < 0.05 was considered as statistically significant.

## Results

### Glucose and S100B increased Steap4 protein expression in MES13 cells

**Figure 1:**
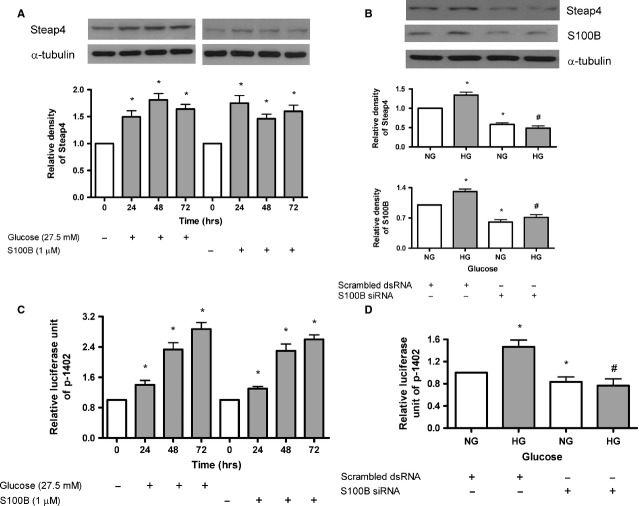
Time-dependent effects of high glucose or S100B on Steap4 protein expression and Steap4 gene transcription in MES13 cells. Cells were exposed to normal glucose (NG, open bars), high glucose (HG, closed bars) or S100B (1 μM, closed bars). Steap4 or S100B protein expression was measured by immunoblotting and was normalized to that of α-tubulin. Steap4 gene transcription was measured by transient transfection of the porcine Steap4 promoter plasmid p-1402. (A) Time-dependent (24–72 hrs) effects of HG (left panel) or S100B (right panel) on Steap4 protein expression. (B) Effects of transient transfection of S100B siRNA on HG-induced Steap4 protein expression at 48 hrs. (C) Time-dependent (24–72 hrs) effects of HG (left panel) or S100B (right panel) on Steap4 gene transcription. (D) Effects of transient transfection of S100B siRNA on HG-induced Steap4 gene transcription at 48 hrs. Data were expressed as the means ± SEM of three independent experiments. *: *P* < 0.05 *versus* open bar. #: *P* < 0.05 *versus* HG alone.

As shown in Figure1A, both HG (27.5 mM) and S100B (1 μM) increased Steap4 protein expression at 24–72 hrs. Moreover, S100B siRNA (but not scrambled siRNA) attenuated HG-induced Steap4 protein expression at 48 hrs (Fig.1B). As shown in Figure1C, both HG and S100B (1 μM) increased Steap4 gene transcriptional activity at 24–72 hrs. Moreover, S100B siRNA (but not scrambled siRNA) attenuated HG-induced Steap4 gene transcriptional activity at 48 hrs (Fig.1D).

### HG increased cell membrane, but not cytosolic Steap4 protein expression

**Figure 2:**
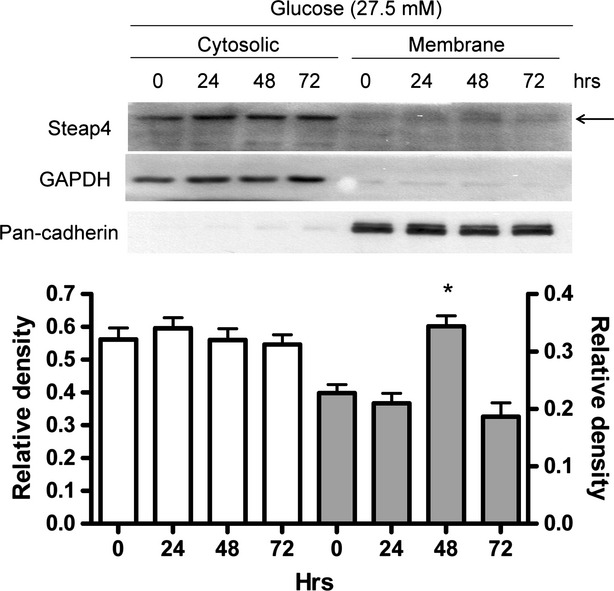
Time-dependent effects of high glucose on cytosolic and cell membrane Steap4 protein expression in MES13 cells. Cells were exposed to high glucose (27.5 mM) for 24–72 hrs. Cells were pelleted and the cytosolic and membrane protein were fractionated by the CNMCS compartmental protein extraction kit. GAPDH was used as a cytosolic marker whereas pan-cadherin was used as a cell membrane marker. Expression of Steap4 protein was measured by immunoblotting and was normalized to that of GAPDH in the cytosol or normalized to that of pan-cadherin in the cell membrane.

Because Steap4 is located in cell membrane, cytosol, Golgi apparatus and endoplasmic reticulum 12,13, it was measured in cytosolic and membrane proteins fractionated by the CNMCS compartmental protein extraction kit. We found that HG increased cell membrane, but not cytosolic, Steap4 protein expression at 48 hrs 3(Fig.2).

### HG increased protein-protein interaction between Steap4 and S100B in MES13 cells

**Figure 3:**
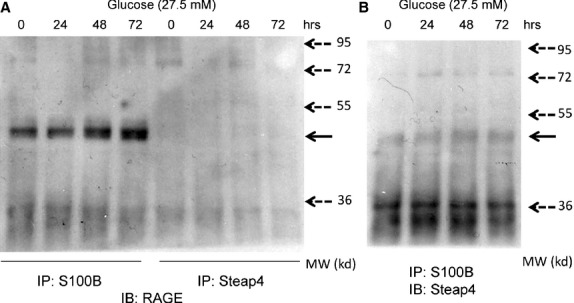
Time-dependent effects of high glucose on protein-protein interaction among RAGE, S100B and Steap4. Cells were exposed to high glucose (27.5 mM) for 24–72 hrs. (A) Immunoprecipitated (IP) S100B or Steap4 was immunoblotted (IB) with RAGE and electrophoresed. Solid arrow: RAGE, broken arrows: molecular weight (MW) markers. (B) Immunoprecipitated (IP) S100B was immunoblotted (IB) with Steap4 and electrophoresed. Solid arrow: Steap4, broken arrows: MW markers.

Because S100B increased Steap4 while S100B is a ligand of RAGE, we measured protein-protein interaction between RAGE and Steap4. We found that HG increased interaction between RAGE and S100B, but there was no interaction between RAGE and Steap4 (Fig.3A). HG also increased interaction between Steap4 and S100B (Fig.3B).

Liquid chromatography-mass spectrometry (LC-MS)/MS 26 of immunoprecipitated S100B was used to identify protein-protein interaction partners of S100B. We found that S100B protein interacted with Steap4, Myh9 (myosin, heavy chain 9), Myh11, Myh14 and α-tropomyosin 1.

### JNK, PI3K and JAK2-STAT3 are required for S100B-induced Steap4 protein expression and gene transcription

**Figure 4:**
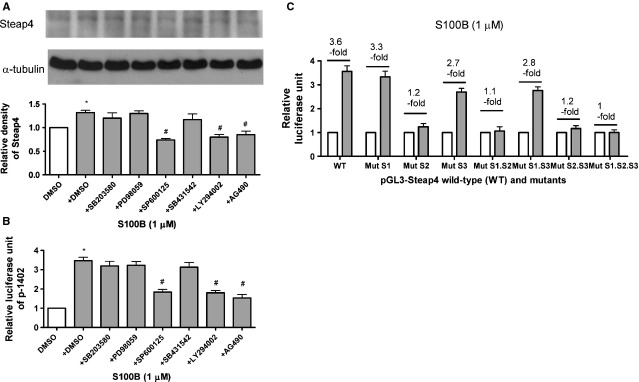
JNK, PI3K and JAK2-STAT3 are required for S100B-induced Steap4 protein expression and gene transcription. Cells were exposed to S100B (1 μM) for 48 hrs. (A) Steap4 protein expression was measured by immunoblotting and was normalized to that of α-tubulin. SB203580 (a p38 kinase inhibitor, 10 μM), PD98059 (an ERK1/2 inhibitor, 10 μM), SP600125 (a JNK inhibitor, 10 μM), SB431542 (a type I TGF-β receptor inhibitor, 10 μM). LY294002 (a PI3K inhibitor, 10 μM) or AG490 (a JAK2 inhibitor, 10 μM) was administered. (B) Steap4 gene transcription was measured by transient transfection of the porcine Steap4 promoter plasmid p-1402. (C) The role of STAT3 in S100B (1 μM)-induced Steap4 protein expression was tested by using the wild-type (WT) and the serially mutated Steap4 promoter constructs (Mut S1, Mut S2, Mut S3, Mut S1.S2, Mut S1.S3, Mut S2.S3 and Mut S1.S2.S3). Data were expressed as the means ± SEM of three independent experiments. *: *P* < 0.05 *versus* open bars. #: *P* < 0.05 *versus* lane 2.

We found that SP600125 (a JNK inhibitor), LY294002 (a PI3K inhibitor) and AG490 (a JAK2 inhibitor), but not SB203580 (a p38 kinase inhibitor), PD98059 (an ERK1/2 inhibitor) or SB431542 (a type I TGF-β receptor inhibitor), attenuated S100B (1 μM)-induced Steap4 protein expression (Fig.4A) or Steap4 gene transcriptional activity (Fig.4B) at 48 hrs.

We tested the role of STAT3 in S100B (1 μM)-induced Steap4 protein expression by using the WT and the serially mutated Steap4 promoter constructs (Mut S1, Mut S2, Mut S3, Mut S1.S2, Mut S1.S3, Mut S2.S3 and Mut S1.S2.S3) 20. We found that S100B (1 μM)-induced a 3.6-fold activation of WT at 48 hrs (Fig.4C). Although mutations in site 1 (Mut S1) and site 3 (Mut S3) had a minor effect, a mutation in site 2 (Mut S2) resulted in a marked decrease in activation at 48 hrs (Fig.4C). Double and triple mutants confirmed the results of single mutants.

### Overexpression of Steap4 attenuates HG or S100B-induced collagen IV, fibronectin and COX2 protein expression

**Figure 5:**
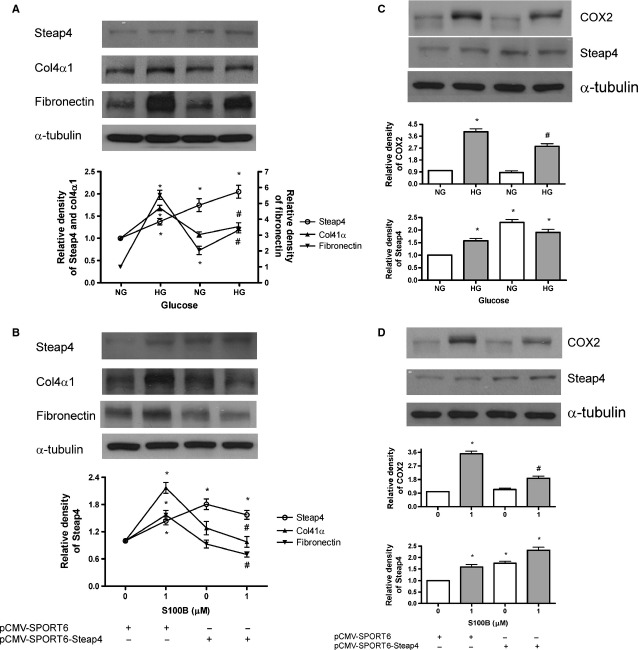
Effects of overexpressing Steap4 on high glucose or S100B-induced Steap4, collagen IV, fibronectin and COX2 protein expression. Cells were exposed to normal glucose (NG, open bars) or high glucose (HG, closed bars) or S100B (1 μM, closed bars) for 48 hrs. Protein expression of collagen IV (col4α1), fibronectin and COX2 was measured by immunoblotting and was normalized to that of α-tubulin. Steap4 overexpression was achieved by transient transfection of the pCMV-SPORT6-Steap4 expression plasmid (2 μg). (A) Effects of overexpressing Steap4 on HG-induced Steap4, collagen IV and fibronectin protein expression. (B) Effects of overexpressing Steap4 on S100B (1 μM)-induced Steap4, collagen IV and fibronectin protein expression. (C) Effects of overexpressing Steap4 on HG-induced COX2 protein expression. (D) Effects of overexpressing Steap4 on S100B (1 μM)-induced COX2 protein expression. Data were expressed as the means ± SEM of three independent experiments. *: *P* < 0.05 *versus* lane 1. #: *P* < 0.05 *versus* lane 2.

As shown in Figure5, pCMV-SPORT6-Steap4 (but not the empty vector) attenuated HG (Fig.5A) or S100B (1 μM; Fig.5B)-induced collagen IV and fibronectin protein expression at 48 hrs. Finally, pCMV-SPORT6-Steap4 (but not the empty vector) attenuated HG (Fig.5C) or S100B (1 μM; Fig.5D)-induced COX2 protein expression at 48 hrs.

### Overexpression of Steap4 attenuates HG or S100B-induced TGF-β bioactivity and gene transcriptional activity

**Figure 6:**
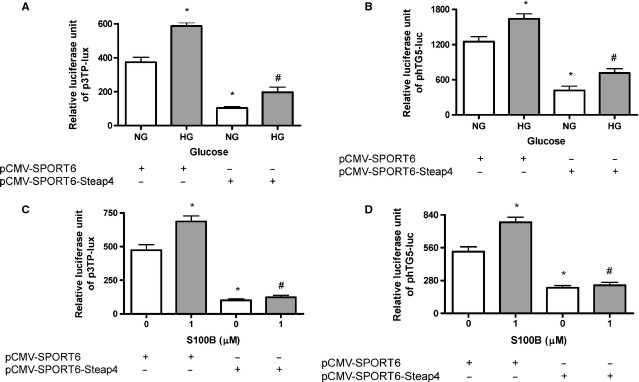
Effects of overexpressing Steap4 on high glucose or S100B-induced TGF-β bioactivity and TGF-β transcriptional activity. Cells were exposed to normal glucose (NG, open bars), high glucose (HG, closed bars) or S100B (1 μM, closed bars) for 24 hrs. Steap4 overexpression plasmid (2 μg) and TGF-β reporter plasmid (phTG5-luc and p3TP-lux, 200 ng) were co-transfected into cells. (A) Effects of overexpressing Steap4 on HG-induced-TGF-β bioactivity. (B) Effects of overexpressing Steap4 on HG-induced-TGF-β promoter activity. (C) Effects of overexpressing Steap4 on S100B (1 μM) induced-TGF-β bioactivity. (D) Effects of overexpressing Steap4 on S100B (1 μM) induced-TGF-β promoter activity. Data were expressed as the means ± SEM of three independent experiments. *: *P* < 0.05 *versus* lane 1. #: *P* < 0.05 *versus* lane 2.

As shown in Figure6, pCMV-SPORT6-Steap4 (but not the empty vector) attenuated HG (Fig.6A) or S100B (1 μM; Fig.6C)-induced TGF-β bioactivity at 24 hrs. Moreover, pCMV-SPORT6-Steap4 (but not the empty vector) attenuated HG (Fig.6B) or S100B (1 μM; Fig.6D)-induced TGF-β gene transcriptional activity at 24 hrs.

### Overexpression of Steap4 attenuates S100B-induced ERK1/2, Akt, STAT3 and Smad2/3

**Figure 7:**
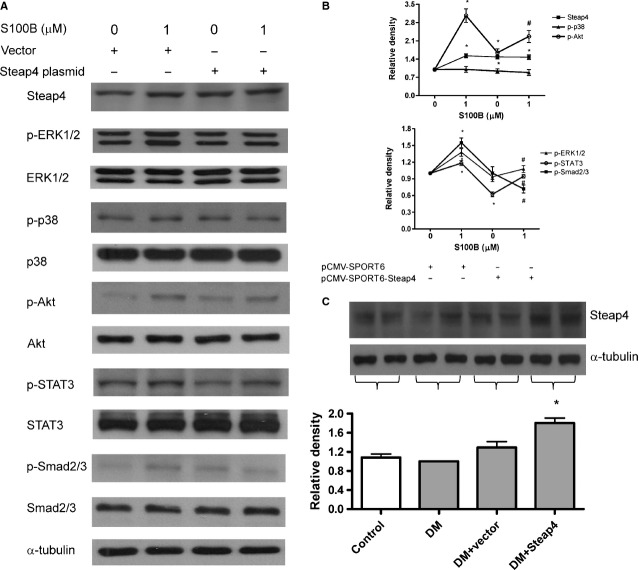
Effects of overexpressing Steap4 on S100B-induced signalling in cells and effects of overexpressing Steap4 on Steap4 protein expression in diabetic mice. Cells were exposed to S100B (1 μM) for 2 hrs. Steap4 overexpression was achieved by transient transfection of the pCMV-SPORT6-Steap4 expression plasmid. (A) Protein expression of Steap4, p-ERK1/2, ERK1/2, p-p38, p38, p-Akt, Akt, p-STAT3, STAT3, p-Smad2/3 and Smad2/3 was measured by immunoblotting and was normalized to that of α-tubulin. (B) Densitometry of (A). *: *P* < 0.05 *versus* lane 1. #: *P* < 0.05 *versus* lane 2. (C) Renal cortex was removed on week 8, homogenized and extracted protein was immunoblotted for Steap4 in control, diabetic (DM), vector-treated (DM + vector) and Steap4 plasmid-treated (DM + Steap4) mice. *: *P* < 0.05 *versus* lane 2. Data were expressed as the means ± SEM of three independent experiments.

As shown in Figure7A and B, S100B (1 μM) activated ERK1/2, Akt, STAT3 and Smad2/3, but not p38 kinase, at 2 hrs. Moreover, pCMV-SPORT6-Steap4 (but not pCMV-SPORT6) attenuated S100B (1 μM)-induced ERK1/2, Akt, STAT3 and Smad2/3 at 2 hrs.

### Overexpressing Steap4 attenuated glomerular fibronectin expression and some of S100B-induced effects in STZ-diabetic mice

**Figure 8:**
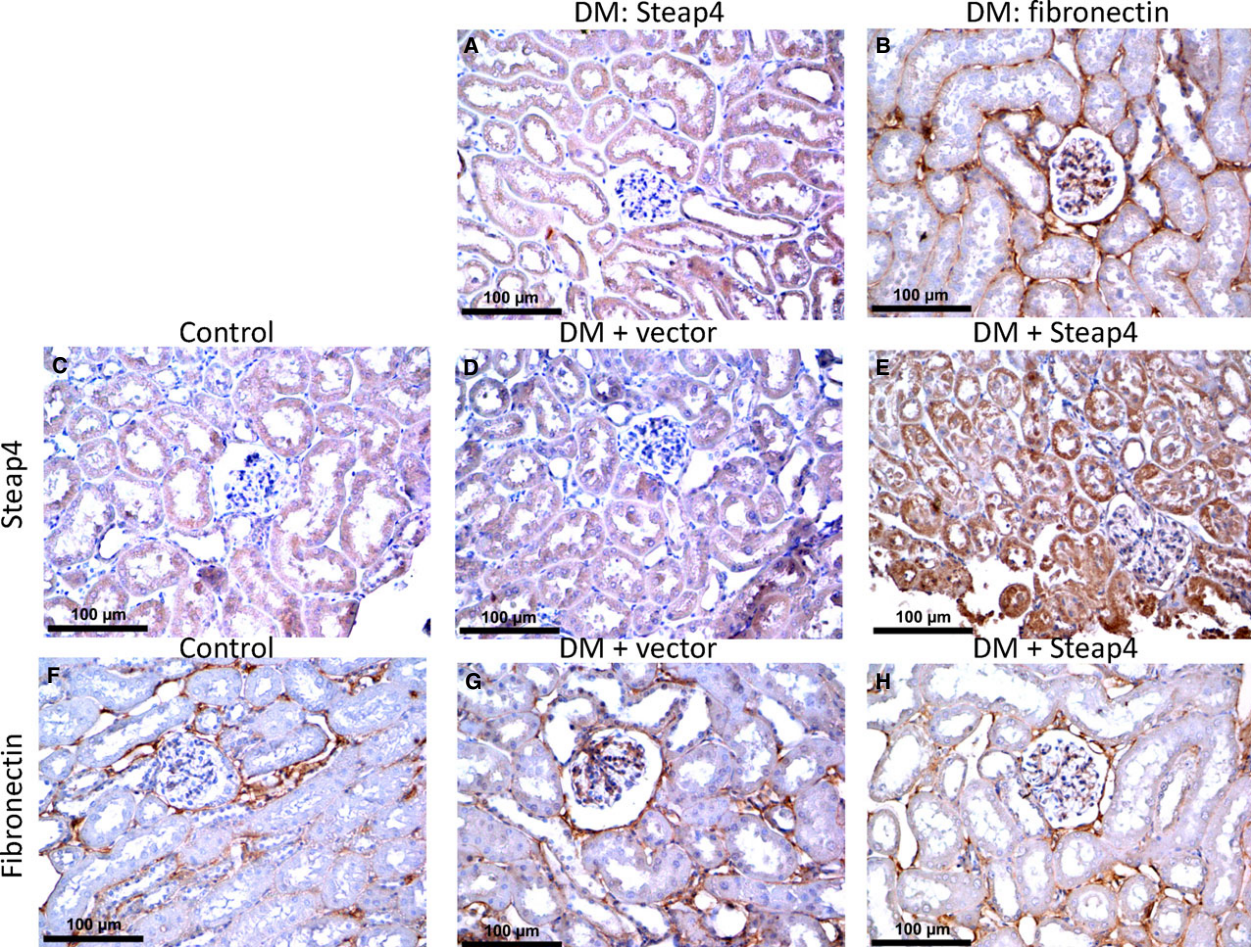
Overexpressing Steap4 attenuated glomerular fibronectin expression in streptozotocin-diabetic mice. Streptozotin-diabetic (DM) mice were given weekly intravenous injection of the pCMV-SPORT6 empty plasmid (DM + vector) or the pCMV-SPORT6-Steap4 expression plasmid (DM + Steap4). Kidneys were removed on week 8, perfused and immersed in 4% paraformaldehyde and kidney slices were embedded in the paraffin block and cut into 3-μm sections for immunohistochemical study. Steap4 (A) and fibronectin (B) expression in a DM mouse was shown. Steap4 expressions in a control (C), DM + vector (D) and DM + Steap4 (E) mouse were shown. Fibronectin expressions in a control (F), DM + vector (G) and DM + Steap4 (H) mouse were shown.

To corroborate the *in vitro* findings, STZ-diabetic mice were given weekly intravenous injection of pCMV-SPORT6-Steap4 expression plasmid. At week 8, blood glucose levels were 155 ± 3.3 mg/dl, 540 ± 2.5 mg/dl, 544 ± 17 mg/dl and 510 ± 17 mg/dl for control, diabetic, vector-treated diabetic and pCMV-SPORT6-Steap4-treated diabetic mice, respectively. Figure7C shows the results of renal cortical expression of Steap4. Note that diabetes and vector did not affect whereas Steap4 overexpression increased Steap4 protein expression. Figure8 shows the results of immunohistochemistry in one control mouse, one diabetic mouse, one pCMV-SPORT6-treated diabetic mouse and one pCMV-SPORT6-Steap4-treated diabetic mouse at 8 weeks. We found that glomerular and tubular Steap4 expression was not affected in the diabetic mouse (Fig.8A) whereas glomerular and tubular Steap4 expression was increased in the pCMV-SPORT6-Steap4-treated (Fig.8E), but not the pCMV-SPORT6-treated (Fig.8D), diabetic mouse. In contrast, glomerular fibronectin expression was increased in the diabetic mouse (Fig.8B) whereas glomerular fibronectin expression was attenuated in the pCMV-SPORT6-Steap4-treated (Fig.8H), but not pCMV-SPORT6-treated (Fig.8G), diabetic mouse.

As shown in Figure S1, vector-treated diabetic mice had increased glomerular and tubular expression of S100B, TGF-β and type IV collagen, which were attenuated by Steap4 overexpression. As shown in Figure S2, vector-treated diabetic mice had increased tubular expression of COX2, increased glomerular and tubular expression of p-Akt and increased glomerular expression of p-ERK1/2. Steap4 overexpression did not affect COX2 expression, but it attenuated glomerular and tubular expression of p-Akt and attenuated glomerular expression of p-ERK1/2. As shown in Figure S3, vector-treated diabetic mice had increased glomerular and tubular expression of p-Stat3, which were attenuated by Steap4 overexpression. Moreover, liver expression of Steap4 was decreased in vector-treated diabetic mice, which was increased by Steap4 overexpression.

## Discussion

This is the first demonstration that S100B is required for HG-induced Steap4 protein expression in MES13 cells. HG increased interaction between S100B and Steap4 while increasing cell membrane, but not cytosolic, Steap4 protein expression. JNK, PI3K and JAK2-STAT3 are required for S100B-induced Steap4 protein expression. Overexpression of Steap4 attenuated HG or S100B-induced effects. Moreover, overexpression of Steap4 attenuated renal fibronectin, TGF-β, collagen IV, p-Akt, p-ERK1/2 and p-Stat3 expression in diabetic mice.

We found that both HG and S100B increased Steap4 protein expression while HG-induced Steap4 was dependent on S100B. This finding is similar to two previous studies showing that Steap4 is increased in HG-cultured macrophages 18 and decreased in RAGE-knockout mouse adipocytes 27. The finding that Steap4 overexpression attenuated HG-induced effects suggest that HG-induced Steap4 expression may be a compensatory mechanism. This scenario is similar to a previous finding that HG increases renal tubular hepatocyte growth factor expression 28 whereas hepatocyte growth factor overexpression attenuates rat DN 29.

Interestingly, we found that HG increased only cell membrane, but not cytosolic, Steap4 protein expression. Moreover, HG increased protein-protein interaction between Steap4 and S100B. This finding was confirmed by co-immunoprecipitation and LC-MS/MS analysis of immunoprecipitated S100B protein complex. The use of LTQ Orbitrap LC-MS/MS to identify interaction partners of S100B-immunoprecipitates to validate Steap4-S100B protein interaction has several advantages. First, unlike other methods, affinity purification combined with MS can study protein interactions as they occur in the cell 30. Second, a high resolution mass spectrometer (LTQ Orbitrap in this case) and the tandem MS/MS mode can increase mass accuracy of the results 31. Because Steap4 moves from the cytosol to the plasma membrane 12, we propose that the interaction between S100B and Steap4 may increase this trafficking.

S100B has been shown to activate p38 kinase, ERK1/2, JNK, TGF-β, PI3K and STAT3 in mesangial or microglial cells 7,32 while the JAK2-STAT pathway is a downstream signal of the RAGE 33. Thus, the roles of p38 kinase, ERK1/2, JNK, TGF-β, PI3K and JAK2 in S100B-induced Steap4 protein expression were studied. We found that JNK, PI3K and JAK2-STAT3 are required for S100B-induced Steap4 protein expression. These findings are compatible with the fact that Steap4 is induced by TNFα, IL1 and IL6 34,35 because TNFα, IL1 or IL6 activates JNK, PI3K/Akt and STAT3 36,37. Moreover, we found that STAT3 site 2 (−116 bp) on the Steap4 gene promoter is required for Steap4 gene transcription. This finding is similar to a previous study showing that STAT3 is required for IL6-induced Steap4 expression in the liver 20.

In this study, overexpression of Steap4 attenuated HG or S100B-induced collagen IV, fibronectin and COX2 protein expression. These findings complement previous studies showing that overexpression of Steap4 decrease pro-inflammatory cytokines (TNFα and IL6) and suppress atherosclerosis in diabetic mice 18,38. Moreover, we found that overexpression of Steap4 attenuated S100B-induced TGF-β and S100B-activated ERK1/2, Akt, STAT3 and Smad2/3. The suppressive effects of Steap4 on S100B-induced effects may be related to the interaction between Steap4 and S100B because S100B can also have RAGE-independent intracellular effects, such as ERK1/2 and Akt 9.

We found that Steap4 protein expression did not change in diabetic mouse kidneys but was decreased in diabetic mouse liver. These findings differed from our *in vitro* findings but corroborated previous studies showing that Steap4 gene or protein is decreased in diabetic mouse liver 39 and diabetic human adipocytes 40. However, our findings that overexpression of Steap4 attenuated increased renal fibronectin, TGF-β, collagen IV, p-Akt, p-ERK1/2 and p-Stat3 protein expression in diabetic mice corroborated our *in vitro* findings. In contrast, we found that tubular (but not glomerular) expression of COX2 was increased in diabetic mouse renal tubules which was not affected by Steap4 overexpression. Interestingly, a previous study also found that tubular (but not glomerular) expression of COX2 was increased in diabetic mouse kidneys 41.

In conclusion, HG-induced Steap4 is dependent on S100B in MES13 cells. Overexpression of Steap4 attenuated HG or S100B-induced effects despite the facts that HG increased cell membrane, but not cytosolic, Steap4 protein expression. Finally, overexpressing Steap4 attenuated some of S100B-induced pathways in streptozotocin-diabetic mice.

## Supporting Information

**Figure S1** Overexpressing Steap4 attenuated glomerular and tubular expressions of S100B, TGF-β and collagen IV in streptozotocin-diabetic mice.

**Figure S2** Overexpressing Steap4 attenuated renal expressions of p-Akt and p-ERK1/2 in streptozotocin-diabetic mice.

**Figure S3** Overexpressing Steap4 attenuated glomerular and tubular expressions of p-Stat3 in streptozotocin-diabetic mice.
