# Supplementary figures and images for: Steap4 attenuates high glucose and S100B-induced effects in mesangial cells
Source: J Cell Mol Med. 2015 Mar 27;19(6):1234–44. doi: 10.1111/jcmm.12472 (PMC4459839; doi:10.1111/jcmm.12472)

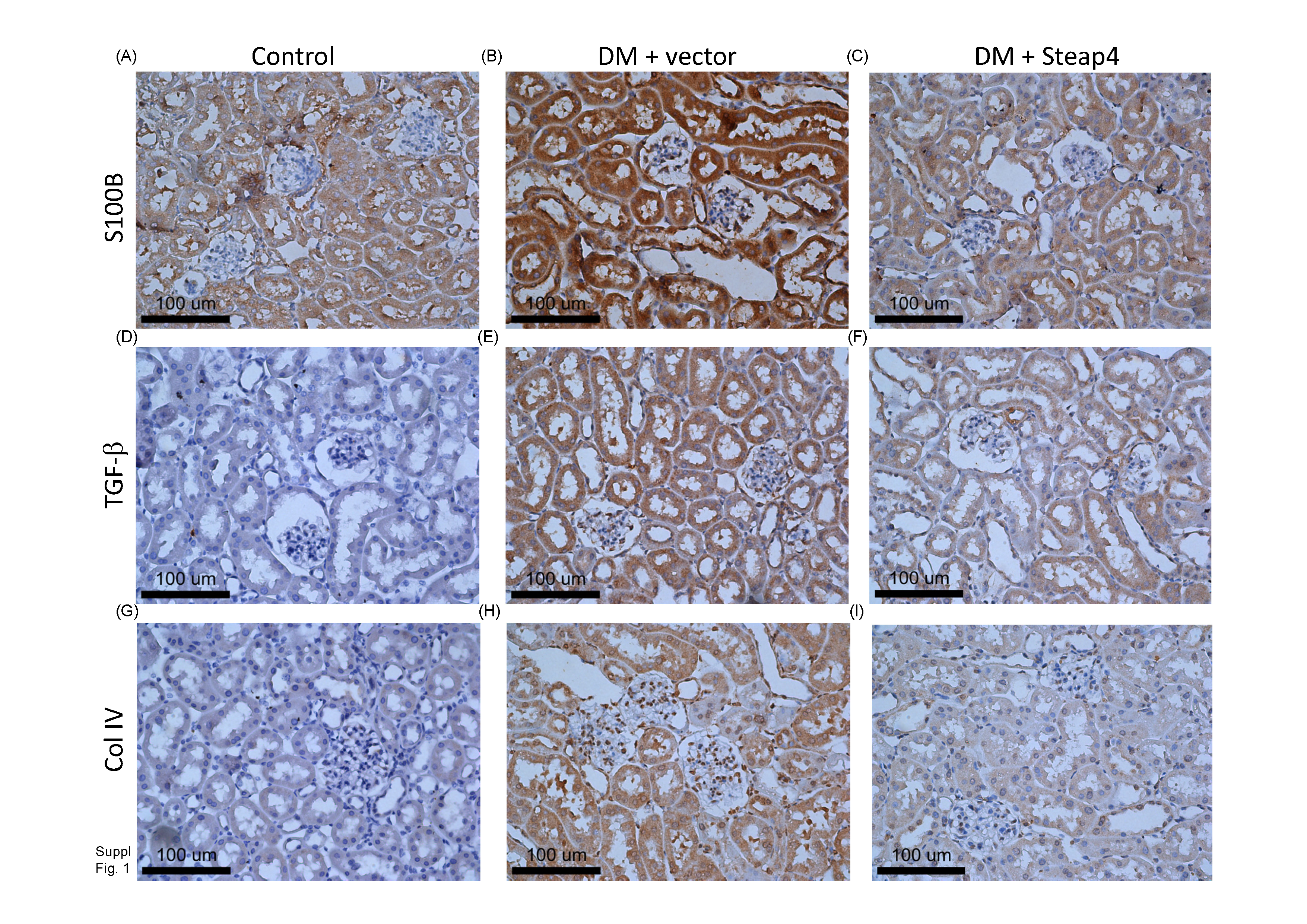

Supplement: Supplementary file 1 [file jcmm0019-1234-sd1.tif]

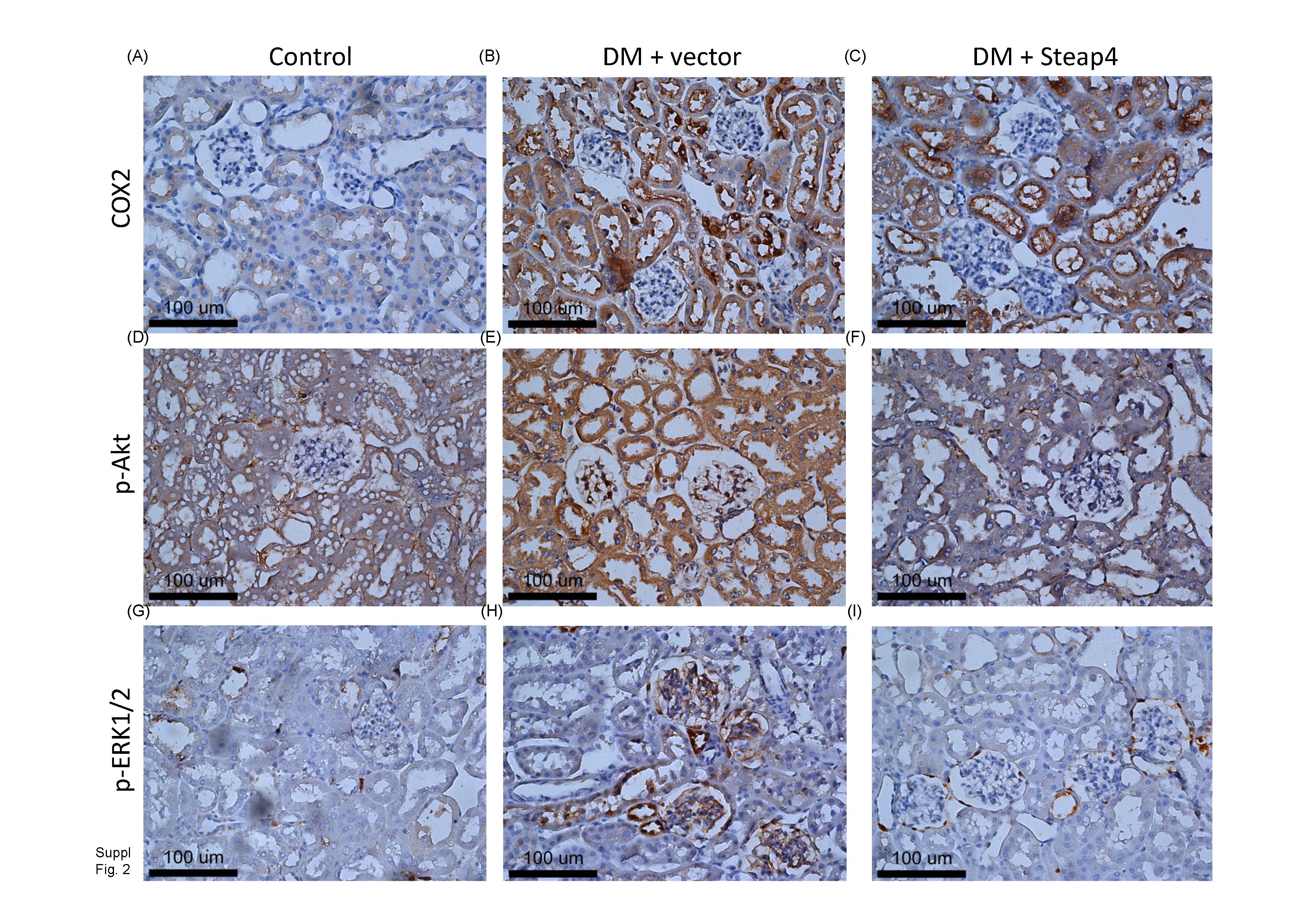

Supplement: Supplementary file 2 [file jcmm0019-1234-sd2.tif]

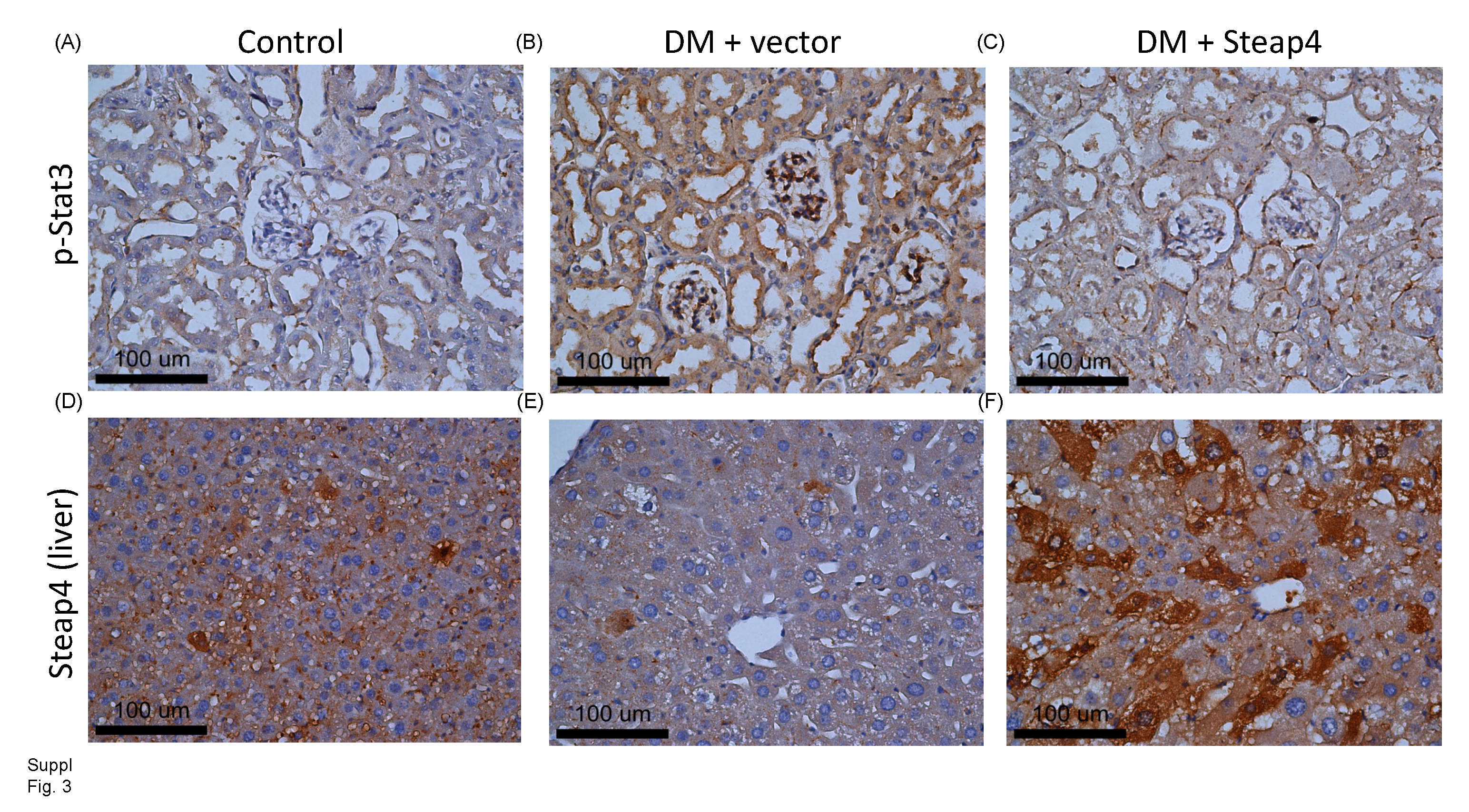

Supplement: Supplementary file 3 [file jcmm0019-1234-sd3.tif]
