# Supplementary material for: Steap4 attenuates high glucose and S100B-induced effects in mesangial cells
Source: J Cell Mol Med. 2015 Mar 27;19(6):1234–44. doi: 10.1111/jcmm.12472 (PMC4459839; doi:10.1111/jcmm.12472)
Supplement: Supplementary file 4 [file jcmm0019-1234-sd4.doc]

**Supplementary Figure Legends**

**Figure 1. Overexpressing Steap4 attenuated glomerular and tubular expressions of S100B, TGF-β and collagen IV in streptozotocin-diabetic mice.**

Streptozotin-diabetic (DM) mice were given weekly intravenous injection of the pCMV-SPORT6 empty plasmid (DM + vector) or the pCMV-SPORT6-Steap4 expression plasmid (DM + Steap4). Kidneys were removed on week 8, perfused and immersed in 4% paraformaldehyde and kidney slices were embedded in the paraffin block and cut into 3-μm sections for immunohistochemical study. S100B expressions in a control (A), DM + vector (B) and DM + Steap4 (C) mouse were shown. TGF-β expressions in a control (D), DM + vector (E) and DM + Steap4 (F) mouse were shown. Collagen IV (Col4α1) expressions in a control (G), DM + vector (H) and DM + Steap4 (I) mouse were shown.

**Figure 2. Overexpressing Steap4 attenuated renal expressions of p-Akt and p-ERK1/2 in streptozotocin-diabetic mice.**

COX2 expressions in a control (A), DM + vector (B) and DM + Steap4 (C) mouse were shown. p-Akt expressions in a control (D), DM + vector (E) and DM + Steap4 (F) mouse were shown. p-ERK1/2 expressions in a control (G), DM + vector (H) and DM + Steap4 (I) mouse were shown.

**Figure 3. Overexpressing Steap4 attenuated glomerular and tubular expressions of p-Stat3 in streptozotocin-diabetic mice.**

p-Stat3 expressions in a control (A), DM + vector (B) and DM + Steap4 (C) mouse were shown. Liver Steap4 expressions in a control (D), DM + vector (E) and DM + Steap4 (F) mouse were shown.
